# *rac-cis*-Cyclo­hexane-1,2-dicarb­oxy­lic acid–isoquinoline (1/1)

**DOI:** 10.1107/S1600536811030613

**Published:** 2011-08-06

**Authors:** Graham Smith, Urs D. Wermuth

**Affiliations:** a Faculty of Science and Technology, Queensland University of Technology, GPO Box 2434, Brisbane, Queensland 4001, Australia

## Abstract

In the crystal structure of the title mol­ecular adduct, C_9_H_7_N·C_8_H_12_O_4_, the two species are ­linked through a carb­oxy­lic acid–isoquinoline O—H⋯N hydrogen bond. These mol­ecular pairs then inter-associate through the second acid group of the *cis*-cyclo­hexane-1,2-dicarb­oxy­lic acid molecules, forming a classic centrosymmetric cyclic head-to-head carb­oxy­lic acid–carboxyl O—H⋯O hydrogen-bonding association [graph-set *R*_2_^2^(8)], giving a zero-dimensional (cluster) structure, consisting of two of each species.

## Related literature

For the structure of racemic *cis*-cyclo­hexane-1,2-dicarb­oxy­lic acid, see: Benedetti *et al.* (1970 ▶). For the structures of the racemic 1:1 ammonium and 2-amino­pyridinium salts of this acid, see: Smith & Wermuth (2011*a* ▶,*b* ▶). For the structure of the 1:1 adduct with 4,4′-bipyridine, see: Bhogala *et al.* (2005 ▶). For hydrogen bonding in carb­oxy­lic acids and graph-set analysis, see: Leiserowitz (1976 ▶); Etter *et al.* (1990 ▶).
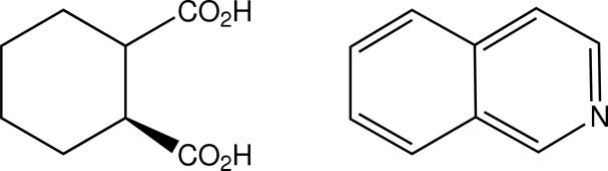


## Experimental

### 

#### Crystal data

C_9_H_7_N·C_8_H_12_O_4_
*M* *_r_* = 301.33
Triclinic, 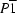

*a* = 6.2459 (3) Å
*b* = 11.4238 (6) Å
*c* = 11.9970 (6) Å
α = 64.082 (5)°
β = 77.793 (4)°
γ = 82.756 (4)°
*V* = 751.95 (7) Å^3^
*Z* = 2
Mo *K*α radiation
μ = 0.10 mm^−1^
*T* = 200 K
0.40 × 0.28 × 0.20 mm

#### Data collection

Oxford Diffraction Gemini-S CCD-detector diffractometer
Absorption correction: multi-scan (*CrysAlis PRO*; Oxford Diffraction, 2010 ▶) *T*_min_ = 0.974, *T*_max_ = 0.990
9094 measured reflections
2952 independent reflections
2463 reflections with *I* > 2σ(*I*)
*R*_int_ = 0.022

#### Refinement

*R*[*F* ^2^ > 2σ(*F* ^2^)] = 0.038
*wR*(*F* ^2^) = 0.090
*S* = 1.02
2952 reflections
207 parameters
H atoms treated by a mixture of independent and constrained refinement
Δρ_max_ = 0.17 e Å^−3^
Δρ_min_ = −0.18 e Å^−3^

### 

Data collection: *CrysAlis PRO* (Oxford Diffraction, 2010 ▶); cell refinement: *CrysAlis PRO*; data reduction: *CrysAlis PRO*; program(s) used to solve structure: *SIR92* (Altomare *et al.*, 1994 ▶); program(s) used to refine structure: *SHELXL97* (Sheldrick, 2008 ▶) within *WinGX* (Farrugia, 1999 ▶); molecular graphics: *PLATON* (Spek, 2009 ▶); software used to prepare material for publication: *PLATON* (Spek, 2009 ▶).

## Supplementary Material

Crystal structure: contains datablock(s) global, I. DOI: 10.1107/S1600536811030613/nk2108sup1.cif

Structure factors: contains datablock(s) I. DOI: 10.1107/S1600536811030613/nk2108Isup2.hkl

Supplementary material file. DOI: 10.1107/S1600536811030613/nk2108Isup3.cml

Additional supplementary materials:  crystallographic information; 3D view; checkCIF report

## Figures and Tables

**Table 1:** Hydrogen-bond geometry (Å, °)

*D*—H⋯*A*	*D*—H	H⋯*A*	*D*⋯*A*	*D*—H⋯*A*
O11—H11⋯O12^i^	0.96 (2)	1.68 (2)	2.6362 (14)	171.7 (18)
O22—H22⋯N2*A*	0.98 (2)	1.69 (2)	2.670 (2)	174.5 (19)

Symmetry code: (i) 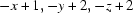.

**Fig. 1.**
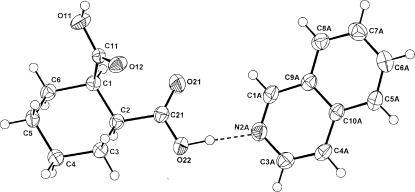
Atom numbering scheme for the two molecules in the title adduct. The inter-species hydrogen bond is shown as a dashed line and displacement ellipsoids are drawn at the 50% probability level.

**Fig. 2.**
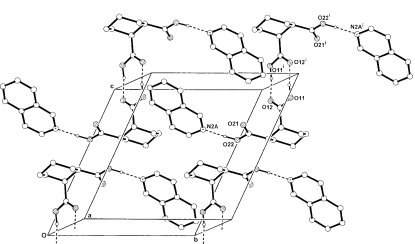
The cyclic carboxylic acid hydrogen-bonding interactions between the acid–base molecular pairs, showing hydrogen bonds as dashed lines. Non-associative H atoms are omitted. For symmetry codes, see Table 1.

## supplementary crystallographic information

### Comment

Although the structure of racemic *cis*-cyclohexane-1,2-dicarboxylic acid is known (Benedetti *et al.*, 1970), together with its 1:1 adduct with 4,4'-bipyridine (Bhogala *et al.*, 2005), there are few examples of salts of this *cis*-acid in the crystallographic literature. We have previously reported the structures of the anhydrous 1:1 ammonium salt (Smith & Wermuth, 2011*a*) and the 2-aminopyridinium salt (Smith & Wermuth, 2011*b*). Our 1:1 stoichiometric interaction of cyclohexane-1,2-dicarboxylic anhydride with isoquinoline in 50% ethanol–water solution gave minor crystals of the 1:1 adduct C_8_H_12_O_4_. C_9_H_7_N, formed in a residual oil, and the structure is reported here.

In the structure of the title adduct (Fig. 1), the two molecular species are interlinked through a carboxylic acid *O*—*H*···N_isoquinoline_ hydrogen bond (Table 1). The molecule pairs then associate through the second acid group, forming a classic centrosymmetric cyclic head-to-head carboxylic acid–carboxyl O—H···O hydrogen-bonding interaction (Leiserowitz, 1976) [graph set *R*^2^_2_(8) (Etter *et al.*, 1990)] giving a zero-dimensional structure (Fig. 2).

### Experimental

The title compound was synthesized by heating a solution of 1 mmol of cyclohexane-1,2-dicarboxylic anhydride and 1 mmol of isoquinoline in 50 ml of 1:1 ethanol–water under reflux for 10 min. After concentration to 30 ml the solution was allowed to evaporate at room temperature, giving a viscous oil which eventually gave minor colourless crystals (m.p. 439–441 K) from which a specimen was cleaved for the X-ray analysis.

### Refinement

The carboxylic acid H atoms were located by difference methods and their positional and isotropic displacement parameters were refined. Other H-atoms were included in the refinement at calculated positions [C—H = 0.93–0.98 Å and with *U*_iso_(H) = 1.2*U*_eq_(aromatic C), or 1.5*U*_eq_(aliphatic C), using a riding-model approximation.

### Crystal data

**Table:**

C_9_H_7_N·C_8_H_12_O_4_	*Z* = 2
*M**_r_* = 301.33	*F*(000) = 320
Triclinic, *P*1	*D*_x_ = 1.331 Mg m^−^^3^
Hall symbol: -P 1	Melting point = 439–441 K
*a* = 6.2459 (3) Å	Mo *K*α radiation, λ = 0.71073 Å
*b* = 11.4238 (6) Å	Cell parameters from 4474 reflections
*c* = 11.9970 (6) Å	θ = 3.3–28.7°
α = 64.082 (5)°	µ = 0.10 mm^−^^1^
β = 77.793 (4)°	*T* = 200 K
γ = 82.756 (4)°	Block, colourless
*V* = 751.95 (7) Å^3^	0.40 × 0.28 × 0.20 mm

### Data collection

**Table:**

Oxford Diffraction Gemini-S CCD-detector diffractometer	2952 independent reflections
Radiation source: Enhance (Mo) X-ray source	2463 reflections with *I* > 2σ(*I*)
graphite	*R*_int_ = 0.022
Detector resolution: 16.077 pixels mm^-1^	θ_max_ = 26.0°, θ_min_ = 3.3°
ω scans	*h* = −7→7
Absorption correction: multi-scan (*CrysAlis PRO*; Oxford Diffraction, 2010)	*k* = −14→14
*T*_min_ = 0.974, *T*_max_ = 0.990	*l* = −14→14
9094 measured reflections	

### Refinement

**Table:**

Refinement on *F*^2^	Primary atom site location: structure-invariant direct methods
Least-squares matrix: full	Secondary atom site location: difference Fourier map
*R*[*F*^2^ > 2σ(*F*^2^)] = 0.038	Hydrogen site location: inferred from neighbouring sites
*wR*(*F*^2^) = 0.090	H atoms treated by a mixture of independent and constrained refinement
*S* = 1.02	*w* = 1/[σ^2^(*F*_o_^2^) + (0.0354*P*)^2^ + 0.20*P*] where *P* = (*F*_o_^2^ + 2*F*_c_^2^)/3
2952 reflections	(Δ/σ)_max_ < 0.001
207 parameters	Δρ_max_ = 0.17 e Å^−^^3^
0 restraints	Δρ_min_ = −0.18 e Å^−^^3^

### Special details

**Table:**

Geometry. Bond distances, angles *etc*. have been calculated using the rounded fractional coordinates. All su's are estimated from the variances of the (full) variance-covariance matrix. The cell e.s.d.'s are taken into account in the estimation of distances, angles and torsion angles
Refinement. Refinement of *F*^2^ against ALL reflections. The weighted *R*-factor *wR* and goodness of fit *S* are based on *F*^2^, conventional *R*-factors *R* are based on *F*, with *F* set to zero for negative *F*^2^. The threshold expression of *F*^2^ > σ(*F*^2^) is used only for calculating *R*-factors(gt) *etc*. and is not relevant to the choice of reflections for refinement. *R*-factors based on *F*^2^ are statistically about twice as large as those based on *F*, and *R*- factors based on ALL data will be even larger.

### Fractional atomic coordinates and isotropic or equivalent isotropic displacement parameters (Å^2^)

**Table:**

	*x*	*y*	*z*	*U*_iso_*/*U*_eq_	
O11	0.70832 (16)	1.07685 (11)	0.86864 (10)	0.0405 (4)	
O12	0.37261 (16)	1.01881 (10)	0.88359 (9)	0.0371 (3)	
O21	0.49576 (18)	0.88369 (10)	0.70388 (12)	0.0476 (4)	
O22	0.17799 (18)	0.95466 (11)	0.63667 (11)	0.0468 (4)	
C1	0.5909 (2)	1.13098 (13)	0.67706 (12)	0.0272 (4)	
C2	0.4067 (2)	1.11424 (13)	0.61893 (12)	0.0272 (4)	
C3	0.1978 (2)	1.19528 (13)	0.63551 (13)	0.0298 (4)	
C4	0.2466 (2)	1.33837 (14)	0.58905 (14)	0.0338 (4)	
C5	0.4233 (2)	1.35310 (13)	0.65186 (13)	0.0314 (4)	
C6	0.6329 (2)	1.27692 (13)	0.62795 (13)	0.0309 (4)	
C11	0.5440 (2)	1.06979 (12)	0.81900 (13)	0.0271 (4)	
C21	0.3661 (2)	0.97179 (14)	0.65964 (13)	0.0308 (4)	
N2A	0.1006 (2)	0.70353 (12)	0.71708 (12)	0.0366 (4)	
C1A	0.2157 (2)	0.61693 (14)	0.79877 (14)	0.0343 (5)	
C3A	−0.0816 (3)	0.66352 (15)	0.69941 (14)	0.0375 (5)	
C4A	−0.1451 (2)	0.53805 (15)	0.76063 (14)	0.0357 (5)	
C5A	−0.0839 (3)	0.31194 (15)	0.92123 (15)	0.0390 (5)	
C6A	0.0362 (3)	0.22778 (16)	1.00839 (16)	0.0434 (5)	
C7A	0.2218 (3)	0.26877 (16)	1.02899 (15)	0.0421 (5)	
C8A	0.2845 (2)	0.39437 (15)	0.96060 (14)	0.0365 (5)	
C9A	0.1621 (2)	0.48468 (14)	0.87014 (13)	0.0304 (4)	
C10A	−0.0255 (2)	0.44335 (14)	0.84987 (13)	0.0303 (4)	
H1	0.72500	1.08880	0.64990	0.0410*	
H2	0.46140	1.14950	0.52810	0.0410*	
H11	0.669 (3)	1.037 (2)	0.959 (2)	0.080 (7)*	
H22	0.158 (3)	0.862 (2)	0.663 (2)	0.078 (6)*	
H31	0.09420	1.18840	0.58900	0.0450*	
H32	0.13100	1.16090	0.72380	0.0450*	
H41	0.29490	1.37600	0.49850	0.0510*	
H42	0.11370	1.38550	0.60730	0.0510*	
H51	0.37080	1.32150	0.74180	0.0470*	
H52	0.45410	1.44450	0.61900	0.0470*	
H61	0.74230	1.28670	0.66960	0.0460*	
H62	0.68990	1.31220	0.53830	0.0460*	
H1A	0.34010	0.64420	0.81040	0.0410*	
H3A	−0.16580	0.72460	0.64320	0.0450*	
H4A	−0.26770	0.51450	0.74370	0.0430*	
H5A	−0.20530	0.28290	0.90850	0.0470*	
H6A	−0.00490	0.14190	1.05510	0.0520*	
H7A	0.30170	0.21020	1.08930	0.0510*	
H8A	0.40850	0.42070	0.97350	0.0440*	

### Atomic displacement parameters (Å^2^)

**Table:**

	*U*^11^	*U*^22^	*U*^33^	*U*^12^	*U*^13^	*U*^23^
O11	0.0362 (6)	0.0521 (7)	0.0326 (6)	−0.0141 (5)	−0.0111 (5)	−0.0120 (5)
O12	0.0331 (6)	0.0448 (6)	0.0305 (5)	−0.0113 (5)	−0.0061 (4)	−0.0105 (5)
O21	0.0450 (6)	0.0329 (6)	0.0715 (8)	0.0055 (5)	−0.0211 (6)	−0.0251 (6)
O22	0.0487 (7)	0.0313 (6)	0.0680 (8)	−0.0050 (5)	−0.0269 (6)	−0.0197 (6)
C1	0.0240 (7)	0.0283 (7)	0.0305 (7)	−0.0012 (5)	−0.0032 (5)	−0.0141 (6)
C2	0.0303 (7)	0.0285 (7)	0.0247 (7)	−0.0036 (6)	−0.0036 (5)	−0.0128 (6)
C3	0.0272 (7)	0.0299 (7)	0.0326 (7)	−0.0030 (6)	−0.0061 (6)	−0.0126 (6)
C4	0.0332 (7)	0.0284 (7)	0.0380 (8)	−0.0008 (6)	−0.0084 (6)	−0.0116 (6)
C5	0.0372 (8)	0.0241 (7)	0.0319 (7)	−0.0056 (6)	−0.0045 (6)	−0.0106 (6)
C6	0.0300 (7)	0.0322 (8)	0.0298 (7)	−0.0085 (6)	−0.0029 (6)	−0.0116 (6)
C11	0.0275 (7)	0.0228 (7)	0.0334 (7)	0.0003 (5)	−0.0081 (6)	−0.0131 (6)
C21	0.0345 (7)	0.0326 (8)	0.0300 (7)	−0.0022 (6)	−0.0053 (6)	−0.0174 (6)
N2A	0.0422 (7)	0.0331 (7)	0.0390 (7)	−0.0029 (5)	−0.0073 (6)	−0.0189 (6)
C1A	0.0326 (8)	0.0379 (8)	0.0420 (8)	−0.0036 (6)	−0.0051 (6)	−0.0256 (7)
C3A	0.0438 (9)	0.0391 (8)	0.0354 (8)	0.0015 (7)	−0.0132 (7)	−0.0190 (7)
C4A	0.0350 (8)	0.0431 (9)	0.0393 (8)	−0.0030 (6)	−0.0101 (6)	−0.0249 (7)
C5A	0.0368 (8)	0.0376 (8)	0.0493 (9)	−0.0062 (6)	−0.0046 (7)	−0.0247 (8)
C6A	0.0498 (10)	0.0316 (8)	0.0468 (9)	−0.0026 (7)	−0.0025 (8)	−0.0172 (7)
C7A	0.0483 (9)	0.0386 (9)	0.0417 (9)	0.0091 (7)	−0.0118 (7)	−0.0201 (7)
C8A	0.0347 (8)	0.0414 (9)	0.0429 (9)	0.0045 (6)	−0.0108 (7)	−0.0262 (7)
C9A	0.0308 (7)	0.0347 (8)	0.0333 (7)	0.0003 (6)	−0.0037 (6)	−0.0225 (6)
C10A	0.0312 (7)	0.0345 (8)	0.0330 (7)	−0.0015 (6)	−0.0025 (6)	−0.0226 (6)

### Geometric parameters (Å, °)

**Table:**

O11—C11	1.3175 (17)	C4—H41	0.9700
O12—C11	1.2237 (17)	C5—H51	0.9700
O21—C21	1.2082 (19)	C5—H52	0.9700
O22—C21	1.3178 (18)	C6—H62	0.9700
O11—H11	0.96 (2)	C6—H61	0.9700
O22—H22	0.98 (2)	C1A—C9A	1.415 (2)
N2A—C3A	1.366 (2)	C3A—C4A	1.361 (2)
N2A—C1A	1.314 (2)	C4A—C10A	1.413 (2)
C1—C2	1.5356 (19)	C5A—C6A	1.361 (3)
C1—C11	1.5081 (19)	C5A—C10A	1.415 (2)
C1—C6	1.543 (2)	C6A—C7A	1.408 (3)
C2—C21	1.519 (2)	C7A—C8A	1.365 (3)
C2—C3	1.532 (2)	C8A—C9A	1.414 (2)
C3—C4	1.527 (2)	C9A—C10A	1.420 (2)
C4—C5	1.5243 (19)	C1A—H1A	0.9300
C5—C6	1.525 (2)	C3A—H3A	0.9300
C1—H1	0.9800	C4A—H4A	0.9300
C2—H2	0.9800	C5A—H5A	0.9300
C3—H31	0.9700	C6A—H6A	0.9300
C3—H32	0.9700	C7A—H7A	0.9300
C4—H42	0.9700	C8A—H8A	0.9300
			
C11—O11—H11	109.3 (12)	C6—C5—H51	110.00
C21—O22—H22	110.7 (12)	C4—C5—H51	109.00
C1A—N2A—C3A	118.09 (14)	H51—C5—H52	108.00
C2—C1—C6	110.04 (11)	C6—C5—H52	110.00
C6—C1—C11	109.54 (12)	C1—C6—H61	109.00
C2—C1—C11	113.10 (11)	C1—C6—H62	109.00
C1—C2—C21	112.12 (11)	C5—C6—H62	109.00
C3—C2—C21	113.55 (11)	H61—C6—H62	108.00
C1—C2—C3	113.23 (12)	C5—C6—H61	109.00
C2—C3—C4	111.43 (11)	N2A—C1A—C9A	124.04 (13)
C3—C4—C5	111.18 (12)	N2A—C3A—C4A	122.78 (15)
C4—C5—C6	110.73 (13)	C3A—C4A—C10A	120.23 (14)
C1—C6—C5	111.33 (11)	C6A—C5A—C10A	120.47 (17)
O11—C11—C1	112.94 (11)	C5A—C6A—C7A	121.01 (17)
O12—C11—C1	124.77 (12)	C6A—C7A—C8A	120.05 (16)
O11—C11—O12	122.29 (13)	C7A—C8A—C9A	120.39 (14)
O22—C21—C2	112.83 (13)	C1A—C9A—C8A	122.95 (13)
O21—C21—C2	123.78 (13)	C1A—C9A—C10A	117.55 (13)
O21—C21—O22	123.33 (16)	C8A—C9A—C10A	119.49 (14)
C2—C1—H1	108.00	C4A—C10A—C5A	124.13 (14)
C11—C1—H1	108.00	C4A—C10A—C9A	117.27 (14)
C6—C1—H1	108.00	C5A—C10A—C9A	118.58 (14)
C1—C2—H2	106.00	N2A—C1A—H1A	118.00
C21—C2—H2	106.00	C9A—C1A—H1A	118.00
C3—C2—H2	106.00	N2A—C3A—H3A	119.00
C2—C3—H31	109.00	C4A—C3A—H3A	119.00
C2—C3—H32	109.00	C3A—C4A—H4A	120.00
C4—C3—H32	109.00	C10A—C4A—H4A	120.00
H31—C3—H32	108.00	C6A—C5A—H5A	120.00
C4—C3—H31	109.00	C10A—C5A—H5A	120.00
C3—C4—H42	109.00	C5A—C6A—H6A	120.00
C5—C4—H41	109.00	C7A—C6A—H6A	119.00
C5—C4—H42	109.00	C6A—C7A—H7A	120.00
H41—C4—H42	108.00	C8A—C7A—H7A	120.00
C3—C4—H41	109.00	C7A—C8A—H8A	120.00
C4—C5—H52	110.00	C9A—C8A—H8A	120.00
			
C3A—N2A—C1A—C9A	−0.3 (2)	C3—C4—C5—C6	57.52 (15)
C1A—N2A—C3A—C4A	−1.4 (2)	C4—C5—C6—C1	−58.31 (15)
C6—C1—C2—C3	−52.58 (14)	N2A—C1A—C9A—C8A	−177.79 (15)
C11—C1—C2—C21	−59.79 (16)	N2A—C1A—C9A—C10A	1.2 (2)
C6—C1—C2—C21	177.35 (11)	N2A—C3A—C4A—C10A	2.1 (3)
C11—C1—C2—C3	70.29 (16)	C3A—C4A—C10A—C5A	177.60 (16)
C2—C1—C11—O11	175.82 (13)	C3A—C4A—C10A—C9A	−1.0 (2)
C2—C1—C11—O12	−4.5 (2)	C10A—C5A—C6A—C7A	−0.5 (3)
C6—C1—C11—O11	−61.03 (16)	C6A—C5A—C10A—C4A	−177.82 (16)
C6—C1—C11—O12	118.67 (16)	C6A—C5A—C10A—C9A	0.8 (2)
C11—C1—C6—C5	−69.79 (14)	C5A—C6A—C7A—C8A	−0.4 (3)
C2—C1—C6—C5	55.15 (14)	C6A—C7A—C8A—C9A	1.0 (3)
C21—C2—C3—C4	−178.03 (11)	C7A—C8A—C9A—C1A	178.30 (16)
C1—C2—C3—C4	52.62 (15)	C7A—C8A—C9A—C10A	−0.7 (2)
C3—C2—C21—O21	−148.08 (14)	C1A—C9A—C10A—C4A	−0.5 (2)
C3—C2—C21—O22	34.64 (16)	C1A—C9A—C10A—C5A	−179.24 (14)
C1—C2—C21—O21	−18.2 (2)	C8A—C9A—C10A—C4A	178.53 (14)
C1—C2—C21—O22	164.55 (12)	C8A—C9A—C10A—C5A	−0.2 (2)
C2—C3—C4—C5	−54.27 (15)		

### Hydrogen-bond geometry (Å, °)

**Table:**

*D*—H···*A*	*D*—H	H···*A*	*D*···*A*	*D*—H···*A*
O11—H11···O12^i^	0.96 (2)	1.68 (2)	2.6362 (14)	171.7 (18)
O22—H22···N2A	0.98 (2)	1.69 (2)	2.670 (2)	174.5 (19)
C3—H32···O12	0.97	2.54	3.1126 (17)	117
C6—H61···O11	0.97	2.53	2.8841 (18)	101

Symmetry codes: (i) −*x*+1, −*y*+2, −*z*+2.

## References

[bb1] Altomare, A., Cascarano, G., Giacovazzo, C., Guagliardi, A., Burla, M. C., Polidori, G. & Camalli, M. (1994). *J. Appl. Cryst.* **27**, 435.

[bb2] Benedetti, E., Pedone, C. & Allegra, G. (1970). *J. Phys. Chem.* **74**, 512–516.

[bb3] Bhogala, B. R., Basavoju, S. & Nangia, A. (2005). *CrystEngComm*, **7**, 551–562.

[bb4] Etter, M. C., MacDonald, J. C. & Bernstein, J. (1990). *Acta Cryst.* B**46**, 256–262. 10.1107/s0108768189012929 2344397

[bb5] Farrugia, L. J. (1999). *J. Appl. Cryst.* **32**, 837–838.

[bb6] Leiserowitz, L. (1976). *Acta Cryst.* B**32**, 775–802.

[bb7] Oxford Diffraction (2010). *CrysAlis PRO* Oxford Diffraction Ltd, Yarnton, England.

[bb8] Sheldrick, G. M. (2008). *Acta Cryst.* A**64**, 112–122. 10.1107/S0108767307043930 18156677

[bb9] Smith, G. & Wermuth, U. D. (2011*a*). *Acta Cryst.* E**67**, o174. 10.1107/S1600536810051883 PMC3050381 21522680

[bb10] Smith, G. & Wermuth, U. D. (2011*b*). *Acta Cryst.* E**67**, o1900. 10.1107/S1600536811025256 PMC3212294 22090951

[bb11] Spek, A. L. (2009). *Acta Cryst.* D**65**, 148–155. 10.1107/S090744490804362X PMC2631630 19171970

